# Trafficking of Siderophore Transporters in *Saccharomyces cerevisiae* and Intracellular Fate of Ferrioxamine B Conjugates

**DOI:** 10.1111/j.1600-0854.2007.00627.x

**Published:** 2007-08-20

**Authors:** Marine Froissard, Naïma Belgareh-Touzé, Marylène Dias, Nicole Buisson, Jean-Michel Camadro, Rosine Haguenauer-Tsapis, Emmanuel Lesuisse

**Affiliations:** 1 Laboratoire Trafic intracellulaire des protéines dans la levure, Département de biologie Cellulaire, Institut Jacques Monod Unité Mixte de Recherche 7592 CNRS-Universités Paris 6 et 7, France; 2 Chimie, Ingénierie Moléculaire et Matériaux d’Angers (CIMMA) Unité Mixte de Recherche 6200 CNRS, Université d’Angers, France; 3 Laboratoire d’Ingénierie des Protéines et Contrôle Métabolique, Département de Biologie des Génomes, Institut Jacques Monod Unité Mixte de Recherche 7592 CNRS-Universités Paris 6 et 7, France

**Keywords:** siderophore, iron, trafficking, *S. cerevisiae*

## Abstract

We have studied the intracellular trafficking of Sit1 [ferrioxamine B (FOB) transporter] and Enb1 (enterobactin transporter) in *Saccharomyces cerevisiae* using green fluorescent protein (GFP) fusion proteins. Enb1 was constitutively targeted to the plasma membrane. Sit1 was essentially targeted to the vacuolar degradation pathway when synthesized in the absence of substrate. Massive plasma membrane sorting of Sit1 was induced by various siderophore substrates of Sit1, and by coprogen, which is not a substrate of Sit1. Thus, different siderophore transporters use different regulated trafficking processes. We also studied the fate of Sit1-mediated internalized siderophores. Ferrioxamine B was recovered in isolated vacuolar fractions, where it could be detected spectrophotometrically. Ferrioxamine B coupled to an inhibitor of mitochondrial protoporphyrinogen oxidase (acifluorfen) could not reach its target unless the cells were disrupted, confirming the tight compartmentalization of siderophores within cells. Ferrioxamine B coupled to a fluorescent moiety, FOB-nitrobenz-2-oxa-1,3-diazole, used as a Sit1-dependent iron source, accumulated in the vacuolar lumen even in mutants displaying a steady-state accumulation of Sit1 at the plasma membrane or in endosomal compartments. Thus, the fates of siderophore transporters and siderophores diverge early in the trafficking process.

Iron can enter *Saccharomyces cerevisiae* cells through two independent pathways: the siderophore uptake pathway and the reductive uptake pathway (1,2). Both pathways are regulated and respond to iron availability (3; for a review, see 4). The reductive pathway (5,6) involves the release of extracellular ferric chelates through reduction at the cell surface by the inducible plasma membrane reductases, Fre1p and Fre2p (7,8). A permease-oxidase complex, Ftr1p and Fet3p, is then involved in translocating iron through the plasma membrane (9,10). The siderophore pathway mediates iron uptake from siderophores – small molecules that bind, solubilize and chelate ferric iron in the environment with high affinity (reviewed in 11). They are synthesized in a non-ribosomal enzymatic process and secreted by bacteria and fungi, but not by *S. cerevisiae*.

The first evidence for the existence of a siderophore transport system in *S. cerevisiae* was provided by the observation that ferrioxamine B (FOB) is efficiently taken up by cells with defective reductive transport of iron (1). Four plasma membrane transporters of the multifacilitator superfamily were later identified as required for the internalization of several ferrisiderophore complexes (12–15). Sit1 (YEL065w), which was first identified as a FOB transporter (12), is the least specific for siderophore substrates, as it can also mediate (with various degrees of efficiency) the transport of various ferrioxamines and ferrichromes (FCH) (4,14). Arn1 (YHL040c) mediates the transport of a specific class of FCHs (14), whereas Taf1 (YHL047c) and Enb1 (YOL158c) seem to be specifically involved in the transport of triacetylfusarinine (TAF) (13) and enterobactin (ENB) (16), respectively.

The intracellular release of iron from siderophores is probably mediated by special reductases, although this remains a matter of debate (17). The release of iron from siderophores is also facilitated by low pH, so special acidic compartments of the cell, such as vacuoles, may also be involved. The details of intracellular ferrisiderophore trafficking, iron release from ferrisiderophores and iron distribution following release remain unclear (reviewed in 11). The intracellular fate of siderophores is an important element in our understanding of the fundamental aspects of iron metabolism. However, it is also important for evaluating the potential efficiency of the ‘Trojan Horse’ approach, which involves combining antibiotics or antifungal drugs with siderophores, enhancing drug accumulation within pathogenic cells (18).

The mechanisms of siderophore transport in *S. cerevisiae* have been thoroughly studied, using Arn1 as a model (17,19,20). It has been shown that Arn1 [carrying a C-terminal hemagglutinin (HA) tag] is sorted directly from the Golgi to the endosomal compartment and is not sorted to the plasma membrane unless the cells are exposed to low concentrations of FCH (19), which acts on a high-affinity C-terminal receptor domain of the protein to control intracellular trafficking of the transporter (20). Homologs of the Arn1 siderophore transporter are present not only in *S. cerevisiae*(Sit1, Taf1 and Enb1) but also in many other fungi, including *Candida albicans*(21–23), *Aspergillus nidulans*(24) and *Schizosaccharomyces pombe*(25,26). It is therefore important to determine whether the regulation of siderophore transporter trafficking described for Arn1 in *S. cerevisiae* can be generalized to other siderophore transporters. We investigated siderophore transporter trafficking for Sit1 and Enb1, two alternative models. We used strains bearing green fluorescent protein (GFP)-tagged versions of the siderophore transporters under the control of the endogenous promoter or under the control of a galactose-inducible promoter (pGAL) to explore potential substrate-induced regulation of the trafficking of these two siderophore transporters.

We also studied the intracellular fate of the siderophore FOB in *S. cerevisiae*, using siderophore analogs. We tried to develop a simple assay for monitoring siderophore fate, using canonical FOB or FOB covalently coupled to the fluorescent moiety nitrobenz-2-oxa-1,3-diazole (NBD). We also investigated the fate of FOB covalently coupled to acifluorfen (AF) (Figure 1), a diphenylether that strongly inhibits protoporphyrinogen oxidase, an essential mitochondrial enzyme (27), to determine whether it would be feasible to use AF coupled to siderophores and taken up by yeast cells as a means of developing new antifungal compounds.

**Figure 1:**
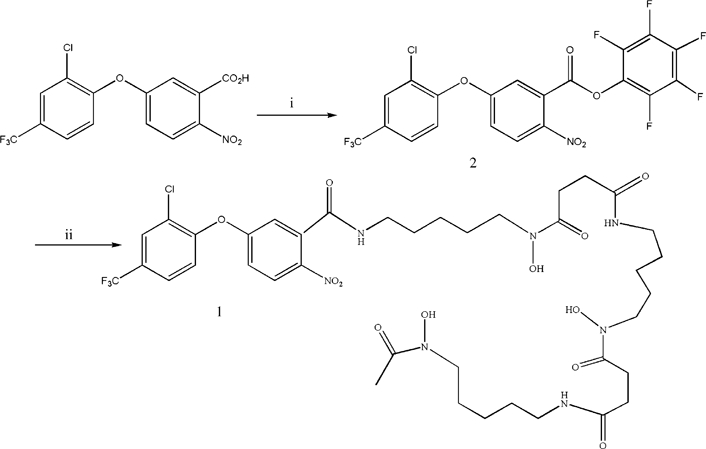
**Synthesis of conjugate AF-DFOB.** Reagents and conditions: (i) AF, pentafluorophenol; DCC, Dicyclohexylcarbodiimide; THF, tetrafuran and (ii) desferal, 2, Et_3_N, THF, 50°C.

## Results

### *Differences in the behaviour of the four siderophore transport systems of* S. cerevisiae

**Figure 2:**
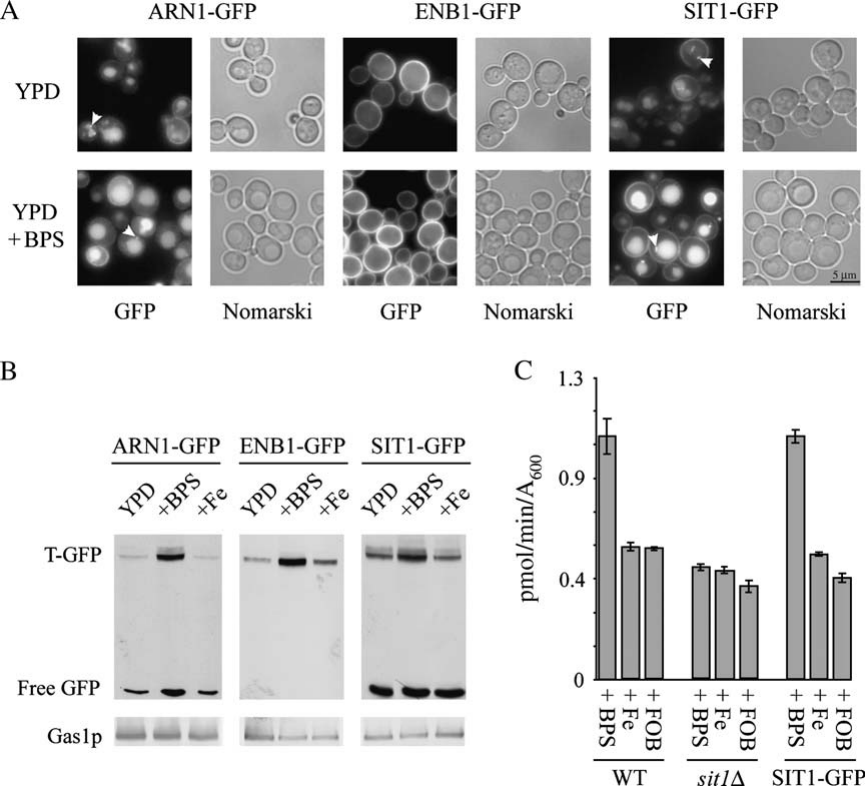
**Synthesis and location of chromosomal GFP-tagged siderophore transporters.**A) Cells expressing chromosomal GFP-tagged siderophore transporters were cultured to midexponential growth phase in complete medium (YPD) or in complete medium supplemented with 200 μm BPS for 4 h (YPD + BPS). Cells were then examined for GFP fluorescence and with Nomarski optics. Arrows indicate round structures possibly corresponding to endosomes. B) Total protein extracts were prepared and analysed by Western blotting for GFP [GFP-tagged transporter (T-GFP) and free vacuolar GFP] and for Gas1p, as a loading control. C) We determined FOB uptake by WT, *sit1*Δ and SIT1-GFP cells cultured to midexponential growth phase in complete medium supplemented with 200 μm BPS, 100 μm iron citrate (Fe) or 100 μm FOB (means ± standard error of the mean from three experiments). WT, wild type.

We checked whether the four siderophore transporters of *S. cerevisiae*(Arn1, Enb1, Sit1 and Taf1) displayed similar intracellular trafficking – sorting from the endosomes to plasma membrane triggered by a specific substrate. We first focused on the relative abundance of the various siderophore transporters and their subcellular distribution and trafficking as a function of growth conditions. We used GFP-tagged versions of the siderophore transporters as an experimental tool. The GFP tag was added to the C-terminus of the siderophore transporters, as previously reported for many plasma membrane transporters with a cytoplasm-oriented C-terminus (28,29). Cells producing chromosomal encoded GFP fusion proteins rather than the original siderophore transporters were used to evaluate the abundance and location of each transporter. We checked that the GFP fusion proteins were active by measuring the rate of iron uptake from FOB, FCH, ENB and TAF by cells expressing Sit1-GFP, Arn1-GFP, Enb1-GFP or Taf1-GFP and comparing these rates to those of cells with unmodified transporters (Figure 2 and S1). We also monitored the GFP-tagged proteins (and vacuolar free GFP) microscopically and by Western blotting (Figure 2). Taf1 is highly specific for TAF, but the Vmax of TAF transport is lower than that of other siderophores transported by Sit1 and Arn1 (30), probably because the concentration of Taf1 is lower than that of other siderophore transporters. Accordingly, the Taf1-GFP fusion protein was not detected by microscopy under any of the growth conditions tested (not shown). The Sit1-GFP, Arn1-GFP and Enb1-GFP transporters were detected in cells grown in both iron-rich (YPD) and iron-deficient (YPD + BPS) conditions (Figure 2A), although they were more abundant under iron-deficient growth conditions, as shown by fluorescence and western immunoblotting (Figure 2B).

**Figure 4:**
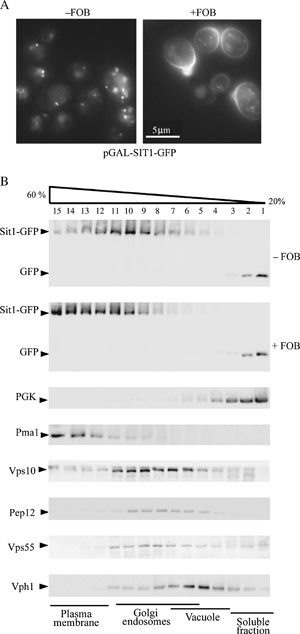
**FOB-dependent localization of Sit1-GFP.**The *sit1Δ* cells transformed with pGAL-SIT1-GFP were cultured overnight in raffinose-containing medium. Sit1-GFP synthesis was induced for 1 h by adding galactose to the medium with (+FOB) or without (−FOB) 10 μm FOB. A) Cells were visualized by fluorescence microscopy with a GFP filter set and B) processed for subcellular fractionation. Cells were lysed and protein extracts were fractionated on a 20–60% sucrose density gradient. Aliquots of the various fractions were analysed by Western immunoblotting for the presence of GFP, PGK (a cytosolic protein), plasma membrane ATPase 1 (Pma1), Vps10 (carboxypeptidase Y receptor, which cycles between the Golgi and late endosome compartments), Pep12 (SNARE protein involved in the fusion of vesicles with late endosomes), Vps55 (transmembrane protein of the late endosomes) and Vph1 (transmembrane subunit of the vacuolar ATPase).

For Arn1-GFP, a significant fraction of the cleaved GFP moiety appeared in the lumen of the vacuole (immunodetectable protein recovered as free GFP) (Figure 2A,B). However, under our experimental conditions, and in contrast to the results obtained by Yun et al. with an HA-tagged version of Arn1 (3), we observed two other types of fluorescent structures: small, intracellular, juxtavacuolar spots, probably corresponding to endosomes (Figure 2A, arrows), and the plasma membrane itself, which was slightly but clearly labelled in some cells, particularly under inducing conditions (low iron concentration; Figure 2A). Hence, most Arn1 is sorted to the vacuolar degradation pathway (19), whereas a small fraction of the protein is sorted to the plasma membrane and may be easier to observe by GFP fusion methods than by indirect immunofluorescence. Enb1-GFP was also observed under all growth conditions (Figure 2A). However, in this case, the fusion protein was systematically restricted to the plasma membrane and was not associated with any other compartment (Figure 2A). Consistent with the exclusive plasma membrane sorting of Enb1, we detected no free GFP by Western blotting (Figure 2B). Some Sit1-GFP was present in internal compartments (Figure 2A, arrows), probably endosomes, as confirmed by subcellular fractionation experiments (see below and Figure 4B). Clear vacuolar staining was observed and was the most prominent signal under noninducing conditions (Figure 2A). Under inducing conditions (low iron concentration), about 90% of the cells also displayed fluorescence associated with the plasma membrane (Figure 2A). We assumed that the GFP fusion proteins behaved similarly to the original proteins, as uptake activities were not affected by GFP tagging, as shown for Sit1 in Figure 2C. Thus, the various siderophore transport systems of *S. cerevisiae* undergo different trafficking processes. Under inducing conditions, Sit1 and Arn1 were detected both in internal compartments and at the plasma membrane; Enb1 was present only at the plasma membrane, and Taf1 was undetectable. We decided to compare the trafficking processes of detectable transporters with radically different patterns of behaviour: Sit1 and Enb1.

### Fate of newly synthesized Sit1 and Enb1 in the absence of their respective substrates

**Figure 3:**
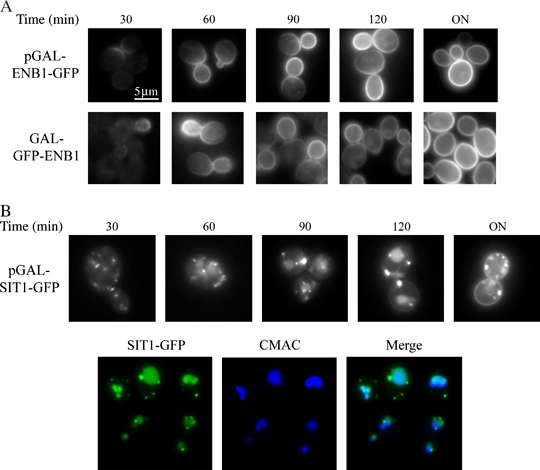
**Kinetics of Enb1 and Sit1 targeting to the plasma membrane and/or in endosomes.**A) *Enb1*Δ cells transformed with pGAL-ENB1-GFP, GAL-GFP-ENB1 cells and B) *sit1*Δ cells transformed with pGAL-SIT1-GFP were cultured to midexponential growth phase with raffinose as the carbon source. Galactose was then added and cells were examined for GFP fluorescence every 30 min for 2 h and after incubation overnight. *Sit1Δ* cells transformed with pGAL-SIT1-GFP, grown in the presence of galactose for 1 h, were stained with CMAC (*Materials and Methods*) to visualize the vacuolar lumen (B).

For monitoring of the fate of newly synthesized fusion proteins, genes encoding GFP fusions of Sit1 and Enb1 were inserted into a centromeric (CEN) plasmid (one to four copies/cell) under the control of the inducible *GAL1* promoter. When this ‘Gal system’ was used to induce transcription in *sit1*Δ and *enb1*Δ cells, galactose was generally added for only short periods (60 min) to prevent the potential mislocalization of proteins because of prolonged overproduction. We checked, by Western blotting, that the level of Sit1 (as GFP fusion) after 1 h of galactose induction was not significantly different from that when the corresponding gene was expressed at the original locus with its endogenous promoter under conditions of iron deprivation (Figure S2). We monitored the intracellular fate of Enb1 and Sit1 and their distribution by fluorescence microscopy after the addition of galactose to cells grown in raffinose-containing medium. Enb1 was targeted exclusively to the plasma membrane, and newly synthesized protein was targeted to the bud (Figure 3A). We checked that the distribution of Enb1 was not affected by the GFP tag, by constructing a chromosome-encoded version of *ENB1* tagged with *GFP* at the N-terminus and placed under the control of the *GAL* promoter. Plasma membrane targeting kinetics were similar for *ENB1* tagged with *GFP* at the N-terminus and for *ENB1* tagged at the C-terminus (Figure 3A).

Sit1 initially accumulated in the Golgi apparatus and endosomal compartments (Figure S3). It was also found in the vacuole after 1 h of synthesis, as shown by colocalization with the vacuolar dye CMAC (Figure 3B). Some plasma membrane staining was observed, but only after overnight overexpression (Figure 3B). These locations were confirmed by subcellular fractionation experiments followed by the western immunoblot analysis of several protein markers (see below and Figure 4B). Thus, newly synthesized Enb1 was targeted directly to the plasma membrane, whereas newly synthesized Sit1 mostly accumulated in Golgi/endosomes and in the vacuole.

### Substrate-induced regulation of Sit1 but not of Enb1 trafficking

Before evaluating the effects of various siderophores on the sorting of Sit1 and Enb1, we determined which siderophores were taken up by these transporters, as the specificity of the transport process may be, to some extent, strain dependent (30). The overexpression of *SIT1-GFP* in *sit1Δ* cells did not increase the uptake of coprogen (CG), TAF or ferric citrate. Instead, it resulted in a massive increase in FOB uptake rate. Ferrichrome and ferricrocin (FC) uptake rates were also significantly increased (Table 1). *ENB1-GFP* overexpression doubled the rate of ENB uptake but had no influence on the uptake of any other iron source (Table 1). Thus, FOB, FCH and FC can reasonably be considered to be substrates of Sit1, whereas ENB is probably the only substrate of Enb1, although this transporter seems to be much less efficient than Sit1, as we previously reported (31). We studied the effect of FOB, the most specific substrate of Sit1, on Sit1-GFP trafficking. Sit1-GFP expression was induced in cells grown overnight on a raffinose-based medium, by incubation with galactose for 60 min in the presence (+FOB) or absence (−FOB) of FOB (10 μm). The distribution of Sit1-GFP was then checked by fluorescence (Figure 4A). In parallel, aliquots of induced cells were disrupted with glass beads, fractionated on sucrose gradients, and the fractions were analysed by western immunobloting for the presence of Sit1-GFP and markers of several compartments (Figure 4B).

**Table 1:** Effect of ENB1-GFP and SIT1-GFP overexpression on the uptake of various iron sources

Strain	WT	ENB1-GFP	SIT1-GFP
Iron source			
FOB	2.99 ± 0.49	4.39 ± 0.56	220.56 ± 13.43
ENB	18.54 ± 1.15	35.48 ± 1.39	12.23 ± 1.15
FCH	31.44 ± 1.12	32.21 ± 1.65	120.18 ± 11.20
FC	32.88 ± 1.48	31.84 ± 4.2	114.51 ± 4.69
CG	4.19 ± 0.14	3.81 ± 0.35	3.4 ± 0.27
TAF	7.55 ± 0.74	8.04 ± 0.32	5.28 ± 0.34
Fe^3+^ (citrate)	14.27 ± 0.85	16.33 ± 0.46	11.03 ± 2.13

Wild-type (WT) cells, Enb1Δ cells transformed with pGAL-ENB1-GFP (ENB1-GFP) and sit1Δ cells transformed with pGAL-SIT1-GFP (SIT1-GFP) were grown overnight in galactose-defined medium, diluted 1:10 in the same medium and grown for a further 5 h. We added 1 μm^55^Fe (as various iron chelates) and determined the amount of iron taken up by the cells (in pmol/A_600_) after 40 min. The results are expressed as means ± standard error of the mean for three experiments.

**Figure 8:**
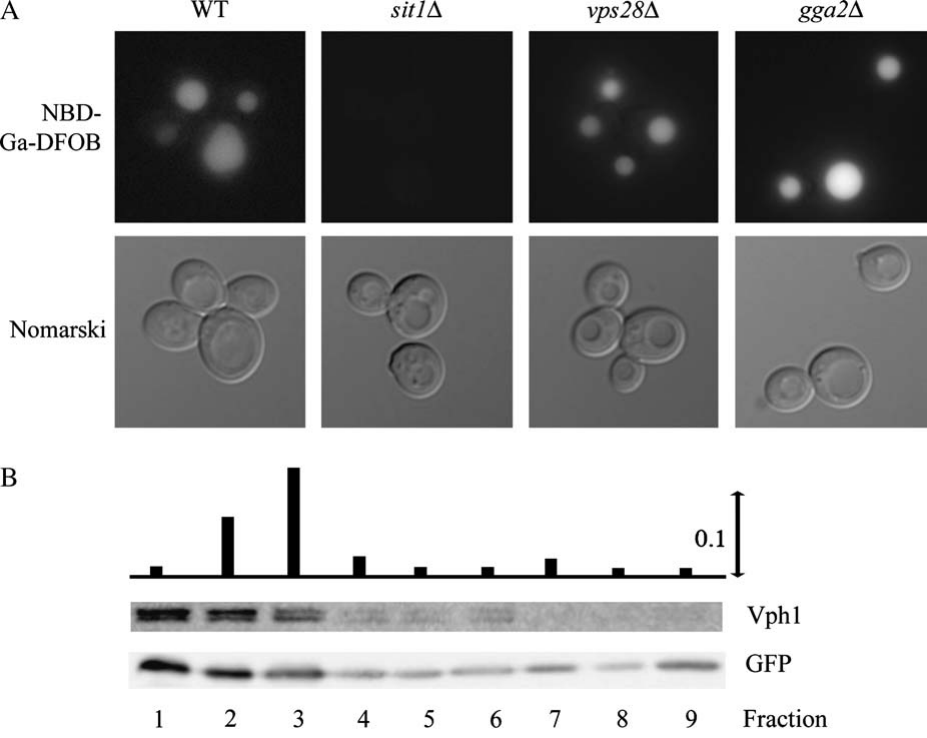
**Intracellular location of FOB-NBD in wild-type (WT) and mutant cells.**A) Wild-type and mutant cells were cultured in glucose-containing medium to midexponential growth phase and were then incubated in the presence of 10 μm Ga-DFOB-NBD for 3 h. The cells were then washed with water and examined for Ga-DFOB-NBD fluorescence using the GFP filter set and with Nomarski optics. B) The *sit1*Δ cells transformed with pGAL-SIT1-GFP were grown overnight in galactose-defined medium supplemented with 100 μm FOB. The whole organellar fraction was collected after protoplast lysis by moderate osmotic shock (*Materials and Methods*). Intact organelles were separated on a discontinuous Ficoll gradient and collected as nine separate fractions. The absorbance of each fraction was measured at 420 nm (maximum absorbance of FOB) after solubilization with 0.1% SDS. The fractions were analysed by Western blotting with an anti-Vph1 (vacuolar marker) and an anti-GFP antibody (free GFP only, corresponding to the cleaved form of Sit1-GFP, present in the vacuolar fractions).

After 1 h of synthesis in the absence of FOB, Sit1-GFP was detected mostly as small dots (−FOB, Figure 4A). These dots may correspond to the Golgi apparatus/endosomal compartments, consistent with cofractionation of internal Sit1-GFP with the Golgi marker Vps10 and the endosomal markers Pep12 and Vps55 (Figure 4B). Faint vacuolar staining was also observed. Accordingly, free GFP was detected by western immunoblotting in light fractions, corresponding to both cytosolic proteins [phosphoglycerate kinase (PGK)] and soluble vacuolar lumen proteins released by cell disruption with glass beads (Figure 4B). After prolonged growth in the presence of FOB and the preparation of intact vacuoles from protoplasts, free GFP was found to colocalize with the vacuolar membrane-bound Vph1 (see Figure 8B). No Sit1-GFP plasma membrane staining was observed in these experimental conditions, but we cannot exclude the possibility that Sit1-GFP was present in trace amounts at the plasma membrane, as a faint signal was detected in subcellular fractions colocalising with the plasma membrane marker plasma membrane ATPase 1 (Pma1) (Figure 4B). Moreover, the main Sit1-GFP signal detected corresponded to fraction 10 of the sucrose gradient (Figure 4B), whereas the main Golgi apparatus/endosomal signals were recovered in fractions 8–9. This observation suggests that Sit1-GFP was not strictly restricted to a ‘pure’ Golgi–endosomal pool. Instead, it may form two different pools: a major endosomal pool and a small plasma membrane pool resulting in a small shift of the main signal on the gradient (Figure 4B).

After 1 h of synthesis in the presence of FOB, Sit1-GFP fluorescence was observed mostly at the plasma membrane, with some intracellular dots (Figure 4A). Accordingly, immunodetected Sit1-GFP was mostly colocalized with the plasma membrane marker Pma1 on sucrose gradients. Some free GFP was also detected, corresponding to a small fraction directly targeted to the Vps/vacuolar pathway or protein subjected to endocytosis (Figure 4B).

**Figure 5:**
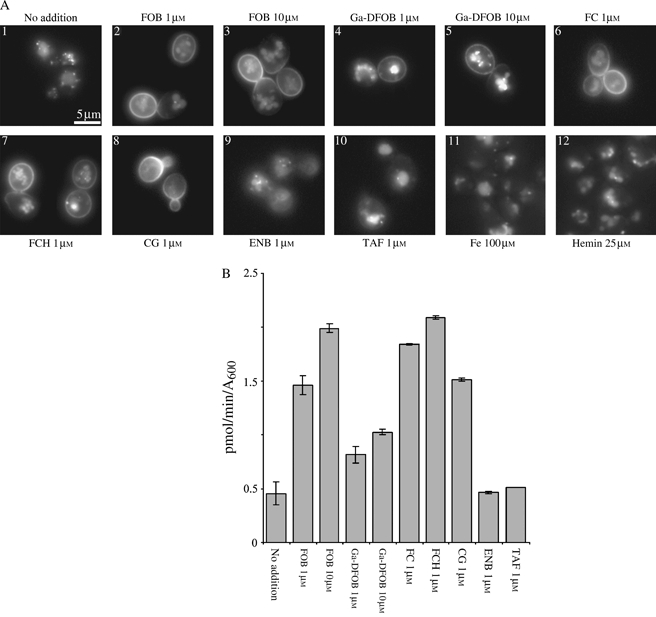
**Siderophore-induced relocation of Sit1-GFP.**The *Sit1*Δ cells transformed with pGAL-SIT1-GFP were cultured to midexponential growth phase in raffinose-containing medium. Sit1-GFP synthesis was induced by incubation with galactose for 60 min and was stopped by adding glucose and incubating for 15 min. The sorting of Sit1-GFP from the endosomes to the plasma membrane was assessed A) by fluorescence microscopy and B) by measuring FOB uptake after incubation with the indicated compound for 30 min. For uptake experiments, the cells were rapidly washed with water and filtered before resuspension in minimal glucose medium containing 1 μm^55^Fe-FOB. The iron content of the cells was determined after 10 min of incubation at 30°C. Uptake values (B) are expressed as means ± standard error of the mean for three experiments.

Thus, Sit1-GFP was mainly sorted to the endosomal/vacuolar pathway when synthesized in the absence of substrate, and mainly targeted to the plasma membrane when synthesized in the presence of substrate. We then checked whether Sit1-GFP initially sorted to internal compartments could subsequently be targeted to the plasma membrane after the addition of FOB or other siderophores. Experiments were performed on *sit1Δ* cells transformed with the pGAL-SIT1-GFP construct and grown overnight on a raffinose-based medium. The cells were incubated with galactose for 60 min for induction. Glucose was added and incubated with the medium for 10–15 min to stop transporter synthesis and a siderophore was then added. The location of the siderophore transporter was determined by assessing fluorescence 30 min after addition of the siderophore, whereas the rate of siderophore transport by the washed cells was measured in parallel experiments. Observations of GFP fluorescence showed that Sit1 was sorted to endosomes and vacuoles after the transient expression of *SIT1-GFP*(Figure 4A, left panel and Figure 5A, panel 1), but rapid, massive translocation to the plasma membrane was observed if the cells were briefly exposed to FOB, the principal substrate of Sit1 (Figure 5A, panels 2 and 3). Plasma membrane sorting from internal compartments was also observed when protein synthesis was inhibited by incubation with cycloheximide for 5 or 15 min before the addition of external FOB. Thus, the external substrate clearly alters the fate of presynthesized Sit1 (Figure S4). The movement of Sit1 to the plasma membrane was accompanied by an increase in the ability of cells to take up FOB (Figure 5B). The gallium analog of FOB [Ga-desferrioxamine (DFOB)] also promoted the movement of Sit1 to the plasma membrane (Figure 5A, panels 4 and 5), although less effectively than FOB itself. The presence of a strong GFP signal in the vacuole lumen indicated that a large proportion of the Sit1 present reached the vacuole for degradation. This effect was observed to a much lesser extent when FOB was the inducer (Figure 5A, panels 2 and 3). Thus, Ga-DFOB was also less effective than FOB at promoting FOB uptake by the washed cells (Figure 5B). Exposure of the cells to two other substrates of Sit1 – FCH and FC – also resulted in the rapid translocation of the protein to the plasma membrane (Figure 5A, panels 6 and 7). Remarkably, CG, which is not a substrate of Sit1 (see below), had the same effect (Figure 5A, panel 8), whereas ENB and TAF had no effect on the distribution of Sit1 (Figure 5A, panels 9 and 10). Other sources of iron, such as ferric citrate and hemin, were also unable to promote the plasma membrane targeting of Sit1 (Figure 5A, panels 11 and 12). The uptake of FOB by the cells was increased in all cases by prior incubation of the cells with a siderophore, promoting the movement of Sit1 to the plasma membrane (Figure 5B). Thus, FOB, FC, FCH and CG all caused Sit1 to move rapidly from internal compartments to the plasma membrane, and this effect occurred at all siderophore concentrations tested (within the range 1–100 μm). Thus, brief exposure of the cells to a siderophore (CG) that is not a substrate of a given transport system (Sit1) may induce this transport system by promoting plasma membrane sorting. This mechanism may have an important adaptive function for cells.

The same experiments were conducted with Enb1-GFP. Galactose induction resulted in the immediate movement of the protein to the plasma membrane (see Figure 3). The kinetics of Enb1 targeting to the plasma membrane were unaffected by the experimental conditions tested (with or without various siderophores in the growth medium; data not shown). Thus, these two siderophore transporters have very different patterns of intracellular trafficking, one with and the other without substrate-induced regulation.

### Fate of Sit1 in wild-type and mutant strains showing high levels of FOB uptake

In a previous study, we showed that several mutants of *S. cerevisiae* deficient in various steps of protein trafficking had a phenotype of high FOB uptake (31). This was notably the case for mutants defective in various stages of Golgi-to-vacuole targeting – Golgi sorting (*gga2Δ*, *arf1Δ*) – or late endosome maturation to multivesicular bodies (MVB) (several *vps* class E mutants). We compared the intracellular fate of Sit1-GFP in wild-type and *sit1Δ* cells, and in one mutant of each of these classes (*gga2Δ*, *vps28Δ*) transformed with pGAL-SIT1-GFP.

**Figure 6:**
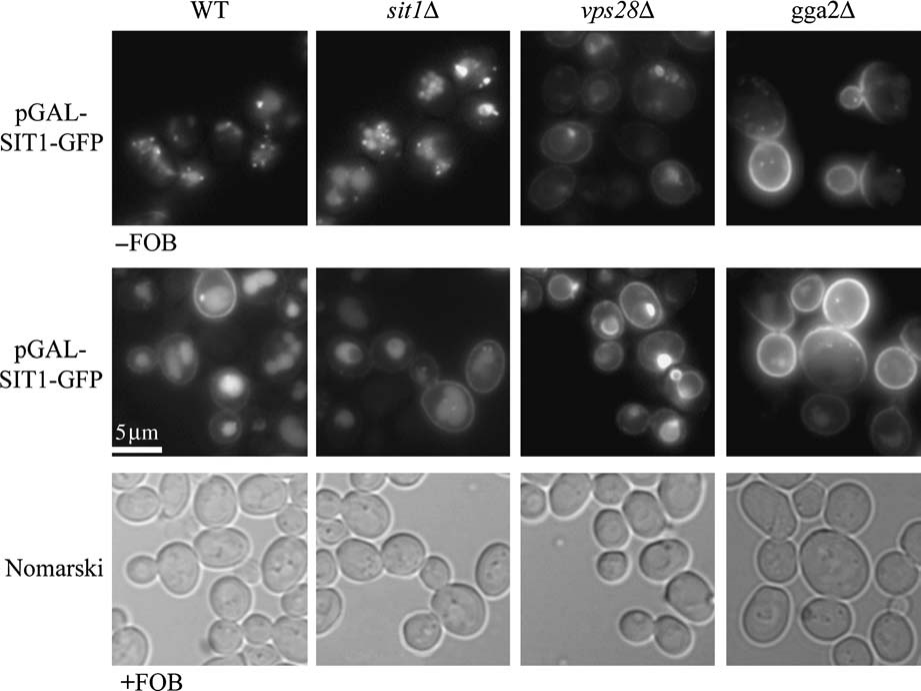
**Sit1-GFP location in wild-type (WT) and mutant cells.**Wild-type and mutant cells, transformed with pGAL-SIT1-GFP, were cultured overnight in raffinose-containing medium to midexponential growth phase. Sit1-GFP synthesis was induced by incubating the cells for 1 h with galactose. Glucose was then added and the culture was incubated for 15 min to stop galactose induction, and GFP fluorescence was monitored either immediately (−FOB) or after a further 30-min incubation of the cells with 1 μm FOB (+FOB).

After galactose induction followed by glucose repression and incubation for 30 min with (+FOB) or without (−FOB) substrate, the steady-state location of Sit1 varied according to the strain examined (Figure 6). As described above, in wild-type and *sit1*Δ cells, Sit1-GFP was targeted to endosomes and the vacuolar lumen in the absence of FOB (Figure 6, −FOB) and was targeted to the plasma membrane in the presence of FOB (Figure 6, +FOB). In the *vps28*Δ mutant, Sit1-GFP accumulated in large juxtavacuolar structures, the class E compartment, and at the vacuolar membrane. The cells also displayed faint plasma membrane staining (Figure 6, −FOB). Plasma membrane staining in the presence of FOB was clearly stronger than that in wild-type cells (Figure 6, +FOB). This observation is consistent with the higher level of FOB uptake observed in these mutant cells (31) and, more generally, with the larger amounts of many transporters at the plasma membrane in the steady state reported for various class E mutants, including *vps28*Δ(32,33). These properties are clearly explained by the sorting defect in these mutants. In *vps28*Δ cells, maturation of the late endosome is compromised and membrane proteins, such as transporters coming from the Golgi compartment or subjected to endocytosis from the plasma membrane, remain at the limiting membrane of a large MVB (known as the class E compartment), rather than being sorted to the internal vesicles of the MVB. This MVB sorting defect leads to the endosome-to-plasma membrane targeting of these proteins (32,34) and to an increase in their steady-state levels at the plasma membrane.

A completely different situation was observed following the production of Sit1-GFP in *gga2*Δ cells. Gga2 is an adaptor protein involved in the Golgi-to-vacuole transport of specific protein substrates (35). We have shown that *gga2*Δ mutant cells have two marked phenotypes in terms of iron metabolism (31): high FOB uptake and the intracellular accumulation of nondissociated FOB. Interestingly, whatever the experimental procedure (−FOB or +FOB), Sit1-GFP was targeted directly to the plasma membrane (Figure 6). Thus, Sit1 is a cargo displaying Gga2-dependent Golgi-to-vacuole targeting. The steady-state distribution of Sit1 at the plasma membrane in *gga2*Δ cells did not prevent the cells from taking up FOB very efficiently (31). This result suggests that FOB is translocated through Sit1 when the transporter is located at the plasma membrane – as would be expected for a ‘classical’ permease – rather than after endocytosis of the transporter, as initially proposed for Arn1 by Kim et al. (20).

### Compartmentalization of intracellular FOB and FOB-NBD in wild-type and mutant cells

**Figure 7:**
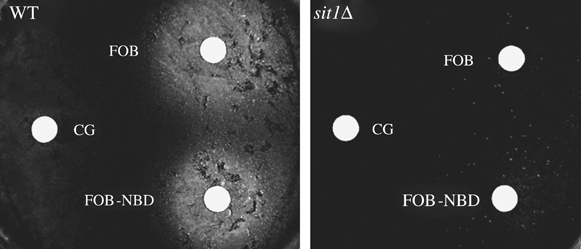
**Sit1-dependent use of FOB-NBD by the cells.**Wild-type (WT) or *sit1*Δ cells were plated on YPD-agar medium supplemented with 1 mm BPS (to prevent reductive iron uptake). Sterile filter paper disks containing siderophores (5 μL of 10 μm CG, FOB or FOB-NBD) were placed on the surface of the agar. Plates were incubated for 2 days at 30°C and photographed. Growing cells appeared as a white halo around the filter papers.

We investigated the intracellular fate of internalized FOB in *S. cerevisiae*, using the NBD fluorescent derivative of FOB (36). We first checked that the cells used the fluorescent derivative of FOB in the same way as native FOB. Desferrioxamine (DFOB)-NBD (7-nitrobenz-2-oxa-1,3-diazole-(D)FOB) (1 μm) fully restored the growth of a *fet3*Δ*fet4*Δ strain cultured on minimal (YNB) medium, as did DFOB (1 μm). After 2 days of incubation in liquid YNB–glucose medium, the *fet3*Δ*fet4*Δ strain had a cell density only 5% that of the wild-type strain, and normal growth was entirely restored when 1 μm DFOB-NBD or DFOB was added to the medium (data not shown). Thus, DFOB-NBD makes iron present in the medium available to cells deficient in reductive iron uptake. Moreover, both FOB and FOB-NBD, unlike CG, restored the growth of a wild-type strain, but not of a *sit1*Δ strain plated on complete agar medium containing a large excess (1 mm) of bathophenanthroline disulfonic acid (BPS) (which prevents reductive iron uptake by the cells; Figure 7). Thus, FOB-NBD is used as an iron source in a Sit1-dependent manner, as is native FOB.

We analysed the intracellular fate of internalized FOB in wild-type and mutant cells using the gallium analog of FOB-NBD rather than ferric FOB-NBD because Ga(III) does not quench the fluorescence of the complex, whereas Fe(III) does. The fluorescent signal obtained with the Ga(III) analog of FOB-NBD was systematically stronger than that obtained with ferric FOB-NBD, although the intracellular location of the fluorescence was the same (data not shown). We also checked that the cells took up the fluorescent analog of FOB as efficiently as FOB itself (see below). Wild-type cells grown in the presence of the gallium analog of FOB-NBD (10 μm) accumulated the fluorescent probe in the vacuole (Figure 8A). No fluorescence was observed in *sit1*Δ cells under the same conditions (Figure 8A), indicating that Sit1 mediated the internalization of the fluorescent siderophore analog, as expected. Sit1 was not expected to reach the vacuole lumen in the *vps28*Δ mutant (see Figure 6). Despite this defect, the fluorescent FOB analog was again found within the vacuole (Figure 8A). Finally, the apparent constitutive location of Sit1 at the plasma membrane in *gga2*Δ cells (see Figure 6) did not prevent the fluorescent FOB analog from accumulating in the vacuole (Figure 8). As FOB-NBD was found essentially in the vacuolar lumen, we checked whether this corresponded to the typical fate of native FOB. For this experiment, we had to overload cells with FOB for the spectrophotometric detection of FOB in the subcellular fractions. Cells transformed with the pGAL-SIT1-GFP plasmid were incubated overnight with galactose in the presence of FOB. Vacuoles were prepared from protoplasts on a Ficoll gradient. Ferrioxamine B-derived visible light absorption and Sit1-GFP-derived free GFP were detected in fractions 1–3, which were enriched in the membrane-bound vacuolar marker Vph1 (Figure 8B). Native FOB was therefore sorted to the vacuoles. Thus, the distribution of Ga-DFOB-NBD reflects the intracellular distribution of the siderophore and can be used as a convenient and rapid method for monitoring the intracellular fate of siderophores.

Our observations clearly indicate that the siderophore transporter (Sit1) and the siderophore itself (FOB) do not follow the same intracellular routes. In this particular case (Sit1/FOB), the fate of the transporter and that of the siderophore may be dissociated early in the process of siderophore uptake. This hypothesis requires confirmation by further experiments, but our data clearly suggest that intracellular FOB is stored in the vacuole, whatever the fate of the FOB transporter. This conclusion is further supported by the experiments using FOB-AF (acifluorfen-(D) FOB) described below.

### Compartmentalization of imported FOB-AF

**Figure 9:**
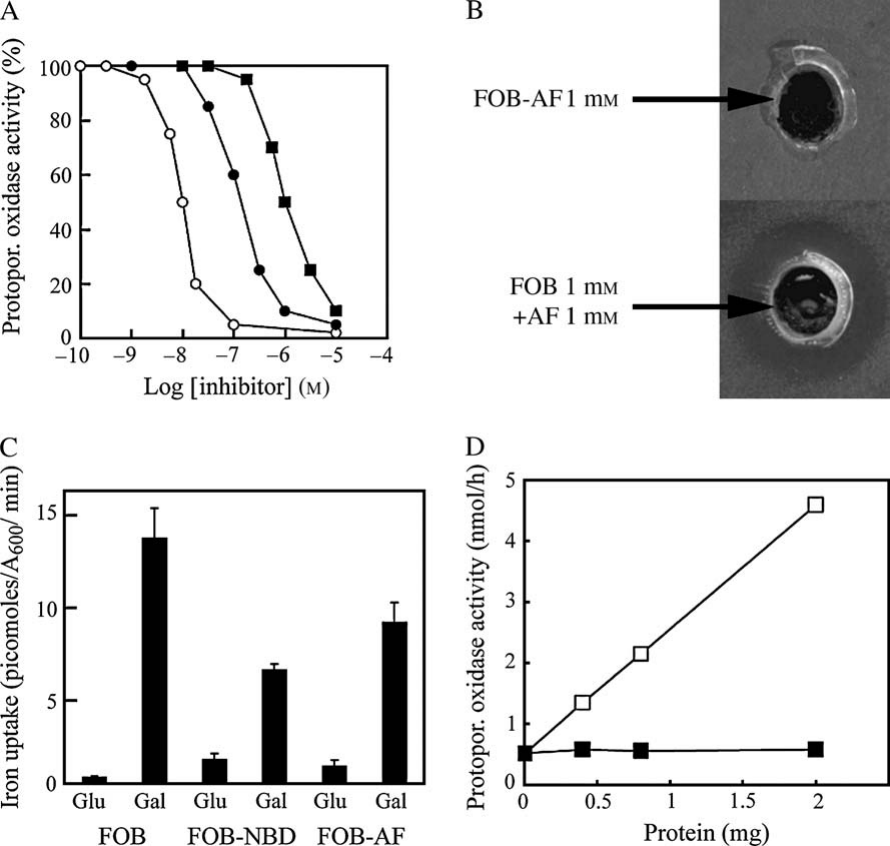
**The AF-FOB conjugate does not reach its target *in vivo*.**A) The IC_50_ values of free AF (○), DFOB-AF (•) and FOB-AF (▪) for protopor. oxidase activity were determined *in vitro* on whole cell extracts. B) Wild-type *S. cerevisiae* cells were plated as a lawn on YPGly agar. Wells were created in the agar with a hole punch, into which 50 μL of 1 mm FOB-AF or 25 μL FOB 2 mm + 25 μL AF 2 mm was dispensed. Cell growth on the plate was examined after 3 days at 30 seconds. It was inhibited only with FOB + AF (inhibition visible as a halo around the bottom well). C) Accumulation of FOB and FOB conjugates in cells overexpressing SIT1. Sit1Δ cells transformed with pGAL-SIT1-GFP were cultured overnight in raffinose-containing medium to midexponential growth phase. We then added glucose (2%) or galactose (2%) and cultured the cells for a further 4 h. ^55^Fe-labelled FOB, FOB-NBD or FOB-AF was then added, and the amount of ^55^Fe accumulated by the cells was determined after 15 min of incubation. Results are expressed as means ± standard error of the mean for six experiments. D) Accumulated FOB-AF inhibits protopor. oxidase *in vitro*. Sit1Δ cells transformed with pGAL-SIT1-GFP were cultured overnight in galactose-containing medium in the presence of 100 μm FOB (□) or 100 μm FOB-AF (▪). The cells were then washed and disrupted with glass beads. The activity of protopor. oxidase was determined on whole cell extracts at various protein concentrations. Protopor., protoporphyrinogen.

Acifluorfen is a powerful inhibitor of protoporphyrinogen oxidase, a mitochondrial enzyme catalysing the formation of protoporphyrin IX from protoporphyrinogen in the heme biosynthetic pathway. We covalently coupled this inhibitor to DFOB, using the same chemical coupling procedure as for Ga-DFOB-NBD (*Methods*) and assessed the inhibitory effect of (D)FOB-AF with respect to AF on protoporphyrinogen oxidase activity *in vitro*. The siderophore drug conjugate displayed weaker inhibitory activity than AF alone (Figure 9A), particularly when iron was bound to the siderophore moiety. This was probably because of steric constraints, as the substrate-binding domain (which is also the inhibitor-binding domain) of protoporphyrinogen oxidase encompasses a narrow active site cavity (37). Despite this limitation, the IC_50_ values measured with DFOB-AF and FOB-AF were nonetheless low enough (about 10^−7^ m and 10^−6^ m, respectively; Figure 9A) for the drug conjugates to be used as potent inhibitors of heme synthesis in yeast. Nevertheless, although AF-methyl efficiently inhibited *S. cerevisiae* growth on agar plates, the siderophore drug conjugate did not (Figure 9B). We then checked whether the cells took up FOB-AF efficiently. Both the fluorescent analog of FOB (FOB-NBD) and the siderophore drug conjugate (FOB-AF) were taken up by the cells through Sit1, although less efficiently than FOB itself (Figure 9C). We cultured cells bearing the *SIT1* gene under control of the *GAL1* promoter with galactose as the carbon source and in the presence of FOB-AF or FOB (100 μm). The washed pellets of cells cultured overnight in these conditions were brownish yellow in colour (the colour of undissociated siderophore), indicating that they had accumulated large amounts of FOB/FOB-AF (data not shown). The cells grown with FOB-AF showed no growth defect (and no porphyrin accumulation), like those grown with FOB (data not shown). However, when these cells were disrupted with glass beads, the protoporphyrinogen oxidase activity measured *in vitro* on cell extracts was completely inhibited in cells grown with FOB-AF (Figure 9D). Thus, protoporphyrinogen oxidase activity was not inhibited *in vivo* in cells that had accumulated enough siderophore drug conjugate to inhibit the enzyme fully *in vitro*, despite considerable dilution of the inhibitor (by about 1:1000) because of cell disruption in suspension buffer. The most probable explanation is that the drug was strictly compartmentalized *in vivo*, such that it could not reach its mitochondrial target unless the cells were completely disrupted. This observation, together with the fluorescence microscopy observations obtained with FOB-NBD, strongly supports the hypothesis that FOB is tightly compartmentalized in the vacuole after its uptake by the cells. It also shows the intrinsic limitation of coupling a drug to a siderophore to generate new antifungal compounds. In this case, FOB-AF cannot be used as an antifungal agent against *S. cerevisiae*, contrary to our expectations.

## Discussion

Our understanding of the mechanisms involved in siderophore transport in yeast has considerably improved over the last few years. One of the most interesting findings to emerge from the study of Arn1 is that this transporter is targeted from the endosome to the plasma membrane, and that this sorting is induced by the substrate of the transporter, FCH, acting on the single extracytosolic C-terminal loop of the protein (19,20). We investigated, in this study, the possibility that this is a property common to other siderophore transporters.

We investigated the intracellular fate of siderophore transporters, using GFP fusion proteins and focusing principally on Sit1 and Enb1 rather than Arn1 and Taf1. Direct fluorescence observations are potentially more sensitive than the indirect immunofluorescence methods used in most studies of Arn1 trafficking, but there is a risk that the GFP tag might alter the behaviour of the fused proteins. We checked that the cells producing GFP transporter fusions took up siderophores in the same way as cells expressing untagged transporters. We used the strong *GAL1* promoter, which can be regulated, to monitor the intracellular fate of transporters, particularly in the presence and absence of substrates, without interference from transcriptional effects. The use of a strong heterologous promoter may generate problems with interpretation, as protein overproduction may lead to mislocalization, but we checked that the amount of protein produced under our experimental conditions (short induction times) did not exceed that with native promoters under inducing conditions (low iron concentration). Taking into account the limitations mentioned above, the main conclusions of our work are as follows. As described for Arn1 (19), the newly synthesized Sit1 protein initially accumulated in endosomal compartments. In contrast, Enb1 protein was directly targeted to the plasma membrane. Direct targeting to the plasma membrane of the FCH transporter of *C. albicans*(*CaSit1*/*CaArn1*) has already been suggested in another study (23) in which the authors demonstrated the plasma membrane location of *CaSit1*/*CaArn1* expressed under the control of its own promoter. We obtained similar results with the transient induction of GFP-tagged *CaSit1*/*CaArn1* placed under the control of the *GAL* promoter and reinserted in two copies at the *CaARN1* locus (Figure S5). Thus, our work shows that different siderophore transporters behave differently in terms of intracellular trafficking.

We found that both Arn1 and Sit1, although mainly present in endosomal compartments, were also targeted to the plasma membrane, to some extent, after expression of the corresponding genes under the control of their native promoters and growth of the cells in the absence of substrate, in iron-deficient conditions. The massive targeting to the plasma membrane of Arn1 and Sit1 from internal compartments was promoted by the addition of siderophores to the medium. However, in contrast to what has been observed for Arn1 (19), we found that the plasma membrane sorting of Sit1 was triggered not only by its specific substrate but also by a siderophore (CG) that is not a substrate of this transporter. This suggests that the sensing domain of the protein may not have the same specificity as the transporting domain, although this point remains to be investigated. All the same, our observations show that it is not necessary to postulate that the substrate must first reach the transporter intracellularly, through a Sit1-independent pathway, before the transporter moves to the plasma membrane. The signal for movement may be sensed at the plasma membrane itself, perhaps through a special high-affinity receptor domain of Sit1 similar to that of Arn1 (20). This may require the presence of small amounts of transporter at the plasma membrane (as observed by fluorescence analysis and sucrose gradient fractionation) even if the transporter is synthesized in the absence of any substrate – for both chromosome-encoded (stable expression) and plasmid-encoded (transient expression) transporters. In contrast to what has been reported for Arn1 and Sit1, we found no evidence of a siderophore-sensing mechanism for Enb1. The transport of different siderophores may involve different uptake mechanisms. Further work is now required to determine exactly how siderophores are transported by the various siderophore transporters of yeast. We also need to determine why structurally similar siderophore transporters may function very differently. The construction of hybrid proteins (e.g. various domains of Arn1 and/or Sit1 fused to other domains of Enb1) should open up new and informative avenues of investigation.

The localization of Sit1-GFP in the presence or absence of FOB in various trafficking mutants provides an explanation for the high FOB uptake phenotype previously observed in many mutants with impaired intracellular protein trafficking (31). Indeed, in cells deficient for MVB sorting (*vps28*), higher than normal levels of Sit1-GFP accumulation at the plasma membrane were observed, even after growth in the absence of FOB, probably because of enhanced endosome-to-plasma membrane sorting, as previously reported for other plasma membrane transporters in *vps* class E mutants (32,33). More strikingly, *gga2Δ* cells, deficient in the clathrin adapter Gga2 and previously shown to display a high level of FOB uptake (31) displayed identical plasma membrane sorting of newly synthesized Sit1-GFP in both the presence and the absence of FOB – Sit1 was therefore not sorted from the Golgi compartment directly to vacuoles in the absence of FOB, as observed in wild-type cells. The siderophore transporter Arn1 has been shown to display partial sorting to the plasma membrane in *gga2Δ* cells, even when synthesized in the absence of its substrate (FCH), under conditions in which wild-type cells display exclusively vacuolar sorting (38). These two siderophore transporters thus extend the list of cargoes displaying Gga2-dependent sorting to the Vps pathway.

We also considered the fate of internalized siderophores (FOB). Internalized FOB undergoes rapid intracellular compartmentalization, as shown by the failure of a FOB drug conjugate (FOB-AF) to reach its mitochondrial target *in vivo*. The observation that siderophores (or at least FOB and FOB conjugates) undergo strict intracellular compartmentalization has major negative implications for the design of new antifungal drugs based on the Trojan Horse approach. Bernier et al. (39) have already reported much lower levels of activity against different *Candida* species for desketoneoenactin-siderophore conjugates than for the unconjugated drug, despite the uptake of the siderophore drug conjugate by cells. They suggested that it might be necessary to release the drug from the conjugate for optimal interaction with the drug target (39). Our study confirms the inefficiency of the Trojan Horse approach and provides an explanation for this observation.

Ferrioxamine B is probably compartmentalized to the vacuole, as deduced from two complementary approaches. We checked that a fluorescent derivative of FOB, FOB-NBD, is used as an iron source in a Sit1-dependent manner. Ferrioxamine B-NBD (or its gallium analog, which displays enhanced fluorescence) was observed to accumulate in the vacuolar lumen of wild-type cells in a Sit1-dependent way. The fate of the fluorescent FOB derivative probably reflects the fate of native FOB: we observed the recovery of native FOB, detected spectrophotometrically, in isolated intact vacuoles prepared from wild-type cells containing Sit1, after gentle protoplast lysis and fractionation on a Ficoll gradient. Conversely, it has been suggested that FCH accumulates in the cytosol, and not in organelles, after its uptake (17). However, these authors fractionated protoplasts by differential centrifugation, which might disrupt vacuoles. The vacuolar accumulation of FOB may be Aft1 dependent, as we previously observed the preferential accumulation of FOB in the late endosome in an AFT1^up^ strain overaccumulating undissociated FOB (30). Perhaps, the intracellular fate (storage and dissociation) of siderophores differs according to the particular siderophore taken up by the cell, as does the mechanism of transport. The structure of ENB (catecholate-type siderophore) is very different from that of FOB (hydroxamate-type siderophore), and it is therefore not unreasonable to assume that these two siderophores might be processed differently by the cell.

As FOB-NBD mimicked the fate of native FOB, and could therefore be considered a powerful and easy-to-use probe, we used it to monitor the intracellular fate of FOB in several trafficking mutants shown to have high levels of FOB uptake in our previous genomic study (39). These mutants displayed enhanced plasma membrane accumulation of Sit1-GFP. Ferrioxamine B-NBD (or its gallium analog) was recovered in the vacuolar lumen of wild-type cells and of *vps28Δ* cells in which Sit1-GFP is located at the plasma membrane and in the class E compartment. Strikingly, *gga2Δ* cells, which have high levels of FOB uptake and in which Sit1-GFP is located exclusively at the plasma membrane, displayed strong vacuolar accumulation of FOB-NBD. The intracellular fate of the transporter (Sit1) therefore appears to be largely independent of the fate of its substrate (FOB). These observations strongly suggest that Sit1 behaves like a classical permease, with FOB being taken up by the transporter at the plasma membrane. If, as shown here for FOB, siderophores accumulate in the vacuole after translocation through the transporter, we need to identify the mechanism of siderophore transport to this organelle. The vacuolar sorting of siderophores is consistent with the notion that the acidic conditions in this organelle might facilitate iron release from the siderophore.

## Materials and Methods

### Yeast strains and growth conditions

The *S. cerevisiae* strains used in this study are shown in Table 2.

**Table 2:** The *S. cerevisiae* strains used

Strain	Relevant genotype	Source
ARN1-GFP	Mata *leu2*Δ*met15*Δ*ura5*Δ*his5*Δ*ARN1-GFP::HIS3*	Clontech
ENB1-GFP	Mata *leu2*Δ*met15*Δ*ura5*Δ*his5*Δ*ENB1-GFP::HIS3*	This study
GFP-ENB1	Mata *leu2*Δ*met15*Δ*ura5*Δ*his5*Δ*Gal-GFP-ENB1-::HIS3*	This study
SIT1-GFP	Mata *leu2*Δ*met15*Δ*ura5*Δ*his5*Δ*SIT1-GFP::HIS3*	Clontech
TAF1-GFP	Mata *leu2*Δ*met15*Δ*ura5*Δ*his5*Δ*TAF1-GFP::HIS3*	This study
BY4741 (wild type)	Mata *leu2*Δ*met15*Δ*ura5*Δ*his3*Δ	Euroscarf
DEY1455 (wild type)	Matalpha *ade2 can1 his3 leu2 trp1 ura3 gal*	(48)
*sit1*Δ	Mata *leu2*Δ*met15*Δ*ura5*Δ*his5*Δ*sit1::kanMX*	Euroscarf
*enb1*Δ	Mata *leu2*Δ*met15*Δ*ura5*Δ*his5*Δ*enb1::kanMX*	Euroscarf
*vps28*Δ	Mata *leu2*Δ*met15*Δ*ura5*Δ*his5*Δ*vps28::kanMX*	Euroscarf
*vam6*Δ	Mata *leu2*Δ*met15*Δ*ura5*Δ*his5*Δ*vam6::kanMX*	Euroscarf
*gga2*Δ	Mata *leu2*Δ*met15*Δ*ura5*Δ*his5*Δ*gga2::kanMX*	Euroscarf
*fet3*Δ*fet4*Δ	Mata *ade6 can1 his3 leu2 trp1 ura3 fet3-2::HIS3 fet4-1::LEU2*	(48)
*CHC-RFP*	Matalpha his3Δ1 leu2Δ0 lys2Δ0 ura3Δ0 YGL206C-RFP::kanMX6	(49)
*SNF7-RFP*	Matalpha his3Δ1 leu2Δ0 lys2Δ0 ura3Δ0 YLR025W-RFP::kanMX6	(49)

*Saccharomyces cerevisiae* strains expressing chromosome-encoded GFP-tagged siderophore transporters were grown at 30°C in 2% glucose in complete medium Yeast extract, bacto Peptone, D-glucose (YPD) supplemented with ferric citrate, BPS or various siderophores, as indicated in the figure legends. Cells transformed with pGAL-ENB1-GFP (p416, ARS/CEN, *URA3*, prom. *GAL1*) or pGAL-SIT1-GFP (p416, ARS/CEN, *URA3*, prom. *GAL1*) were grown at 30°C in minimal medium Yeast Nitrogen Base (YNB) containing 0.67% yeast nitrogen base without amino acids (Difco) and supplemented with the appropriate nutrients. The carbon source was 2% glucose, or 2% galactose plus 0.02% glucose, as indicated in the figure legends. Cells grown overnight in 2% raffinose plus 0.02% glucose to an A_600 nm_ of 0.5 were induced with 2% galactose, which was added to the medium to induce synthesis of the siderophore transporter. Following the addition of this sugar, the cells were incubated for further 60–90 min at 30°C in the presence or absence of FOB, as indicated in the figure legends. In cells used for studies on plasma membrane sorting, transporter synthesis was stopped by adding 2% glucose to the medium. The cells were then incubated for 30 min with the siderophore, at the concentrations indicated in the figure legends.

The *C. albicans* strain used (*CaArn1*Δ::*cat*/*CaArn1*Δ::*hisG*, pABSK-*GAL1-10*-*CaARN1*-*GFP*) was a gift from Dr Y. Wang (23) who constructed it from the CAI4 wild-type strain.

### Synthesis of DFOB conjugates

DFOB-NBD, the fluorescent derivative of DFOB was synthesized as previously described (40).

#### Chemical synthesis of AF-DFOB conjugate 1

The route followed for the synthesis of compound 1 is outlined in Figure 1. Ligand 1 was obtained by a classic two-step procedure. Acifluorfen was activated through DCC-mediated condensation with pentafluorophenol to give the activated ester 2. This ester reacted with DFOB, in the presence of triethylamine, in dimethyl formamide (DMF) to yield 1. All reagents were of analytical grade and were dried and purified when necessary. Thin-layer chromatography (TLC) analyses were carried out on plates coated with silica or alumina gel 60 F254 (Merck). We used 60A silica gel with particles 6–35 or 40–63 μm in size (SDS) or Florisil® 100–200 mesh (ACROS) for column chomatography. Spectra were obtained as follows: ^1^H NMR spectra were recorded at 500 MHz on a Brucker AVANCE DRX 500. The MALDI-TOF-MS spectrum was recorded on a Brucker BIFLEX III. Melting points were determined on a Kofler bank and are uncorrected.

#### 2,3,4,5,6-Pentafluorophenyl-5-(2-chloro-4-trifluoromethylphenoxy)-2-nitrobenzoate (2)

A solution of 5-(2-chloro-4-trifluoromethylphenoxy)benzoic acid (Acifluofen) (1.60 g, 4.44 mmol) in dry tetrahydrofuran (3 mL) was placed in a three-necked round-bottomed flask under nitrogen. A solution of pentafluorophenol (0.82 g, 4.44 mmol) and DCC (0.92 g, 4.44 mmol) in dry tetrahydrofuran (7 mL) was added dropwise to the flask. The mixture was stirred under N_2_ at room temperature for 14 h. The resulting precipitate was removed by filtration, and the filtrate was concentrated to dryness under reduced pressure. The crude product was subjected to chromatography on a silica gel column. Elution with methylene chloride–petroleum ether (80:20) yielded a white solid identified as 2 (2.21 g, 94%). Melting point 106–108°C. ^1^H NMR [500 MHz, dimethyl sulphoxide (DMSO)-*d_6_*]: δ 7.12 [d.d (doublet doublet), 1H, *J*= 9 and 2.7 Hz], 7.30 [d (doublet), 1H, *J*= 8.4 Hz], 7.35 (d, 1H, *J*= 2.6 Hz), 7.65 (d.d, 1H, *J*= 8.5 and 1.8 Hz), 7,84 (d, 1H, *J*= 1.8 Hz), 8.16 (d, 1H, *J*= 9 Hz).

#### DFOB-AF (1)

Desferrioxamine mesylate salt (Desferal®) (700 mg, 1.06 mmol) and pentafluorophenyl ester 2 (728 mg, 1.38 mmol) were dissolved in DMF (18 mL) and triethylamine (1.4 mL) under nitrogen. The reaction mixture was stirred at 50°C for 19 h. The solvent was removed under reduced pressure, and the crude product was purified by column chromatography on silica gel, with methylene chloride–methanol (95:5) as the eluent. The resulting product was washed several times with ethyl acetate and then recrystallized from methanol to yield 462 mg of conjugate DFOB-AF 1 (44%). Melting point 170–172°C. ^1^H NMR (500 MHz, DMSO-d_6_): δ 1.19–1.51 [m (multiplet), 18H], 1.94 [s (singulet), 3H], 2.24 [t (triplet), 4H], *J*= 7.2 Hz, 2.56 (t, 4H, *J*= 6.8 Hz), 2.98 (m, 4H), 3.16 (t, 2H, *J*= 6.6 Hz), 3.44 (t, 6H, *J*= 7 Hz), 7.19 (d.d, 1H, *J*= 8.9 and 2.7 Hz), 7.22 (d, 1H, *J*= 2.6 Hz), 7.48 (d, 1H, *J*= 8.5 Hz), 7.77 (s, 2H), 7.83 (d.d, 1H, *J*= 8.5 and 1.6 Hz), 8.09 (d, 1H, *J*= 8.9 Hz), 8.14 (d, 1H, *J*= 1.6 Hz), 9.60, 9.62 and 9.65 (3 s, 3H). MS (MALDI-TOF): *m/z*926 (M+Na)^+^, 942 (M+K)^+^.

### Strains and plasmid constructions

Yeast strains expressing GFP-tagged siderophore transporters were constructed by inserting the GFP coding sequence in frame with and at the C-terminus of the *ENB1* and *TAF1* genes by homologous recombination (41). ENB1 was also tagged with GFP at its N-terminus and placed under the control of a GAL10 promoter, by the same method (41). The pGAL-ENB1-GFP and pGAL-SIT1-GFP plasmids were obtained by inserting fragments encoding *ENB1* and *SIT1*, respectively, with a C-terminal GFP tag into the p416-GAL vector (42). These fragments were obtained by polymerase chain reaction, using genomic DNA from the chromosomal GFP-tagged strains as templates.

### Siderophore uptake

Coprogen and ENB were purchased from EMC Microcollection GmbH (Germany). Desferri-FCH was purchased from Sigma. Desferrioxamine B is the commercially available mesylate derivative, Desferal (Novartis). Triacetylfusarinine was a gift from Dr H. Haas (Department of Molecular Biology, Medical University of Innsbruck, Austria). The desferri-siderophores were loaded with ^55^Fe (50 mCi/mg). Iron uptake was assayed in microtitration plates (30). Unless otherwise stated, the cells were incubated for various periods of time at 30°C with 1 μm siderophore (final concentration), collected with a cell harvester (Brandel) and washed on the filter with water.

### Subcellular fractionation

Cell fractions were separated by gradient density centrifugation, essentially as previously described by Kolling and Hollenberg (43). The growth of exponentially growing cultures (after galactose induction, glucose stopped, with or without FOB) was arrested by adding 10 mm sodium azide. Cells were harvested by centrifugation, washed once in the presence of 10 mm sodium azide and disrupted by vortexing (four times, for 1 min each) in the presence of glass beads and 0.5 mL of STE10 [10 mm Tris–HCl buffer, pH 7.6 supplemented with 10% wt/wt sucrose, 10 mm ethylenediaminetetraacetic acid (EDTA), 25 mm freshly prepared *N*-ethylmaleimide and protease inhibitors (Complete Cocktail, Roche)]. The extract was transferred to a fresh tube and the beads were washed twice with STE10. The extract and washings were pooled and centrifuged at 3000 × ***g***for 3 min to remove cell debris. An aliquot (750 μL) of the cleared extract was layered onto a 11.1 mL 20–60% linear sucrose gradient made up in Tris–HCl, pH 7.6 supplemented with 10 mm EDTA. Samples were centrifuged for 18 h at 100 000 × ***g***in an SW41 rotor (Beckman). We collected 0.8 mL fractions and precipitated the proteins in these fractions by adding 0.8 mL of 20% trichloroacetic acid (TCA) and incubating on ice for 60 min. Proteins were collected by centrifugation at 13 000 × ***g***for 45 min, resuspended in 10 μL of 1 m Tris base + 40 μL of 2× sample buffer (44) and heated for 10 min at 37°C.

Intact vacuoles were prepared on a discontinuous Ficoll gradient after lysis of the protoplast suspension by moderate osmotic shock, as previously described (45). All the proteins isolated by these fractionation methods were analysed by Western blotting, as described below.

### Yeast cell extracts, SDS–PAGE and Western immunoblotting

Total protein extracts were prepared by the NaOH/TCA lysis technique (44). Proteins were separated by SDS–PAGE, in gels containing 12% tricine, and transferred to nitrocellulose membranes. The membranes were probed with various antibodies, depending on the experiment: monoclonal antibodies against GFP (Roche Diagnostics), Vph1p (Molecular Probes), Vps10p (Molecular Probes), PGK (Molecular Probes) or polyclonal antibodies against Pep12p (kindly provided by K. Bowers and T. Stevens) and Pma1p (kindly provided by C. W. Slayman).

An antibody against the endosomal protein Vps55p was raised in rabbit (46). A mixture of two synthetic peptides (in the second loop and the soluble carboxy-terminus of the protein) was used to immunize two rabbits (Eurogentec, Belgium).

Primary antibodies were detected with horseradish peroxidase-conjugated anti-rabbit or anti-mouse immunoglobulin G secondary antibody (Sigma). Secondary antibodies were detected with an enhanced chemiluminescence kit (ECL, Amersham).

### Fluorescence microscopy

We used an Olympus BY61 microscope equipped with fluorescence and Nomarski optics for our observations and a Spot charge-coupled device camera (SPOT4.05) for image acquisition.

Green fluorescent protein fluorescence was monitored in cells collected from the cultures and examined under the microscope with the fluorescein isothiocyanate (FITC) filter set either directly or after a single wash with water.

For experiments investigating the colocalization of GFP staining with the vacuolar lumen, cell cultures were incubated for 15 min with 5 μm 7-amino-4-chloromethylcoumarin (CMAC) (Cell Tracker Blue, Molecular Probes). The cells were collected and washed twice with water and were then examined under the microscope for GFP staining and blue vacuolar staining [6-diamidino-2-phenylindol-dihydrochlorid (DAPI) filter].

For yeast cell staining with the fluorescent analog of DFOB, DFOB-NBD was loaded with equimolar concentrations of Fe(III) or Ga(III) salts to give FOB-NBD or Ga-DFOB-NBD. Wild-type and mutant cells were cultured overnight in glucose complete medium diluted to an A_600_ of 0.3–0.5 with fresh glucose complete medium supplemented with 10 μm FOB-NBD or Ga-DFOB-NBD. The culture was incubated for 3 h. The cells were then washed twice with water and examined for fluorescence with the FITC filter set and Nomarski optics.

### Assays of growth in response to various iron sources

We investigated iron-dependent growth by plating cells as a lawn on YPD-agar medium supplemented with 1 mm BPS (to prevent reductive iron uptake). The cells were provided with various sources of iron in the form of sterile filter paper disks containing siderophores (5 μL of 10 μm CG, FOB or FOB-NBD). Plates were incubated at 30°C for 2 days and photographed.

Conjugated AF toxicity to cells was evaluated by plating wild-type *S. cerevisiae* cells as a lawn on a YPGly agar medium. We added 50 μL of 1 mm FOB-AF or 25 μL FOB 2 mm + 25 μL AF 2 mm to wells made in the agar with a hole punch. Cell growth on the plate was examined after 3 days at 30°C.

### Activity and inhibition of protoporphyrinogen oxidase

The activity of this enzyme was measured fluorimetrically as previously described (47). IC_50_ values were determined with protoporphyrinogen oxidase purified to homogeneity as previously described (47). Protoporphyrinogen oxidase inhibition by endogenous FOB-AF was assessed on homogenates of cells disrupted with glass beads.

## Supplementary Materials

Figure S1 **Siderophore uptake by cells expressing native transporters (WT) and by cells expressing chromosome-encoded GFP fusion transporters (SIT1-GFP, ARN1-GFP, TAF1-GFP and ENB1-GFP).** The indicated strains were first cultured overnight in YPD medium. Cultures were then diluted 1:10 in the same medium supplemented with 200 μm BPS and incubated for 6 h. The cells were washed with water, resuspended in fresh YPD medium and iron uptake was measured with 1 μm of FOB, FCH, TAF or ENB. Results are expressed as the rate of uptake per A_600_ unit. Means ± standard error from three experiments are shown.

Figure S2 **Comparison of Sit1-GFP levels.** Cells expressing chromosomeencoded Sit1-GFP were cultured to midexponential growth phase in complete medium (YPD). Sit1Δ cells bearing pGAL-SIT1-GFP were grown to midexponential growth phase in raffinose-containing medium. Galactose was then added to the medium and the cultures incubated for 1 or 2 h to induce Sit1-GFP synthesis. Protein extracts were prepared from both strains and analysed by Western blotting for GFP, using a monoclonal anti-GFP antibody.

Figure S3 **Colocalization of Sit1-GFP with CHC1-red fluorescent protein (RFP) and Snf7-RFP.** CHC1-RFP (late Golgi marker) and SNF7-RFP (late endosome marker) strains transformed with pGAL-SIT1-GFP were grown overnight in raffinose-containing medium. Galactose was then added and the culture incubated for 1 h to induce Sit1-GFP expression. The distributions of Sit1-GFP, CHC1-RFP and Snf7-RFP were compared, using the GFP and rhodamine filter sets. Arrows indicate colocalized structures and arrowheads, structures that were not colocalized. Snf7- RFP expression leads to the accumulation of a class E compartment (large late endosome apposed to the vacuole) in which Sit1-GFP is trapped.

Figure S4 **Siderophore-induced relocation of Sit1-GFP independent of protein synthesis.** The sit1Δ cells transformed with pGAL-SIT1-GFP were cultured to midexponential growth phase in raffinose-containing medium. Sit1-GFP synthesis was induced by incubation with galactose for 60 min. Protein synthesis was blocked by adding cycloheximide (CHX, 10 μg/mL) to the medium either together with galactose (pannel 1; control to ensure that protein synthesis was immediately blocked with this concentration of CHX) or after the galactose incubation period (panels 2 and 3). In the latter case, cells were incubated with CHX for 5 min (panel 2) or 15 min (panel 3) before adding FOB (1 μm) and incubating the cells for a further 15 min. Sit1-GFP sorting was assessed by fluorescence microscopy, using the GFP filter set.

Figure S5 **Constitutive targeting of CaSit1/CaArn1 from *C. albicans* to the plasma membrane.** Cells with chromosomal GFP tags were cultured to midexponential growth phase in raffinose minimal medium. CaSit1/CaArn1-GFP synthesis was induced by incubation with galactose for 90 min. GFP fluorescence was assessed by fluorescence microscopy, using the GFP filter set.
