# Supplementary figures and images for: Trafficking of Siderophore Transporters in Saccharomyces cerevisiae and Intracellular Fate of Ferrioxamine B Conjugates
Source: Traffic. 2007 Aug 20;8(11):1601–16. doi: 10.1111/j.1600-0854.2007.00627.x (PMC2171038; doi:10.1111/j.1600-0854.2007.00627.x)

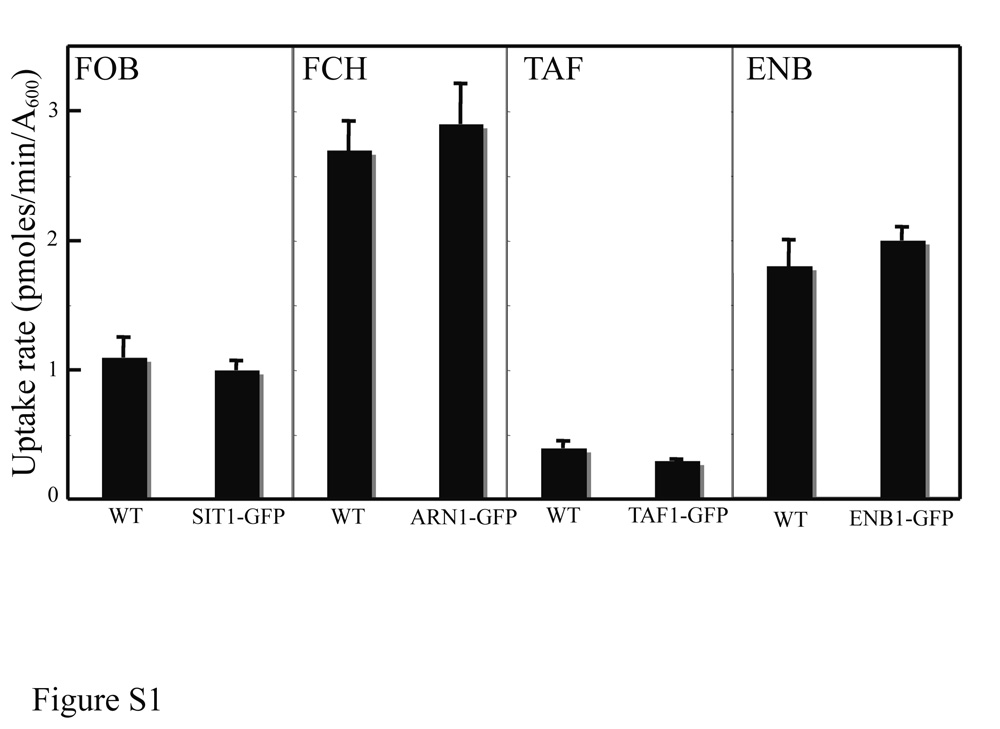

Supplement: Figure S1 — Siderophore uptake by cells expressing native transporters (WT) and by cells expressing chromosome-encoded GFP fusion transporters (SIT1-GFP, ARN1-GFP, TAF1-GFP and ENB1-GFP). The indicated strains were first cultured overnight in YPD medium. Cultures were then diluted 1:10 in the same medium supplemented with 200 μm BPS and incubated for 6 h. The cells were washed with water, resuspended in fresh YPD medium and iron uptake was measured with 1 μm of FOB, FCH, TAF or ENB. Results are expressed as the rate of uptake per A600 unit. Means ± standard error from three experiments are shown. [file tra0008-1601-SD1.jpg]

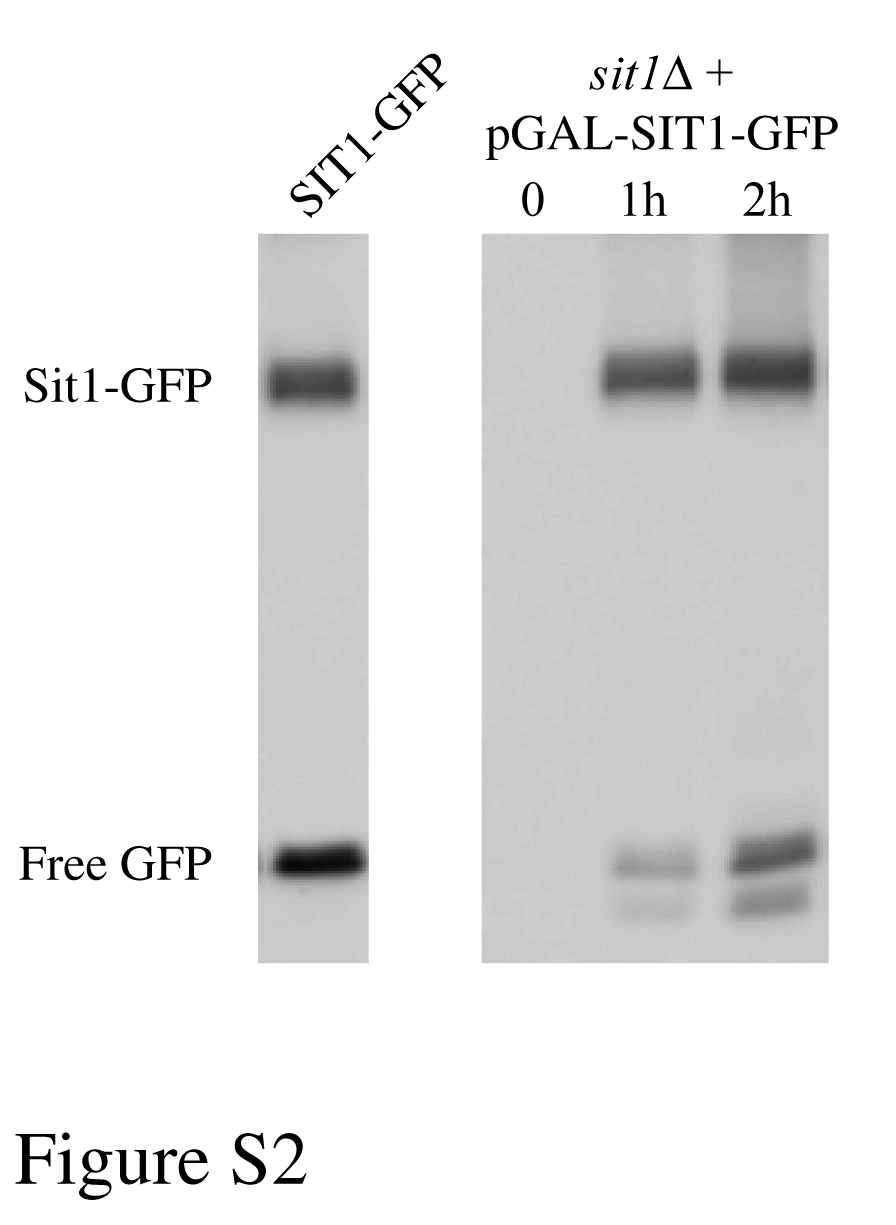

Supplement: Figure S2 — Comparison of Sit1-GFP levels. Cells expressing chromosomeencoded Sit1-GFP were cultured to midexponential growth phase in complete medium (YPD). Sit1Δ cells bearing pGAL-SIT1-GFP were grown to midexponential growth phase in raffinose-containing medium. Galactose was then added to the medium and the cultures incubated for 1 or 2 h to induce Sit1-GFP synthesis. Protein extracts were prepared from both strains and analysed by Western blotting for GFP, using a monoclonal anti-GFP antibody. [file tra0008-1601-SD2.jpg]

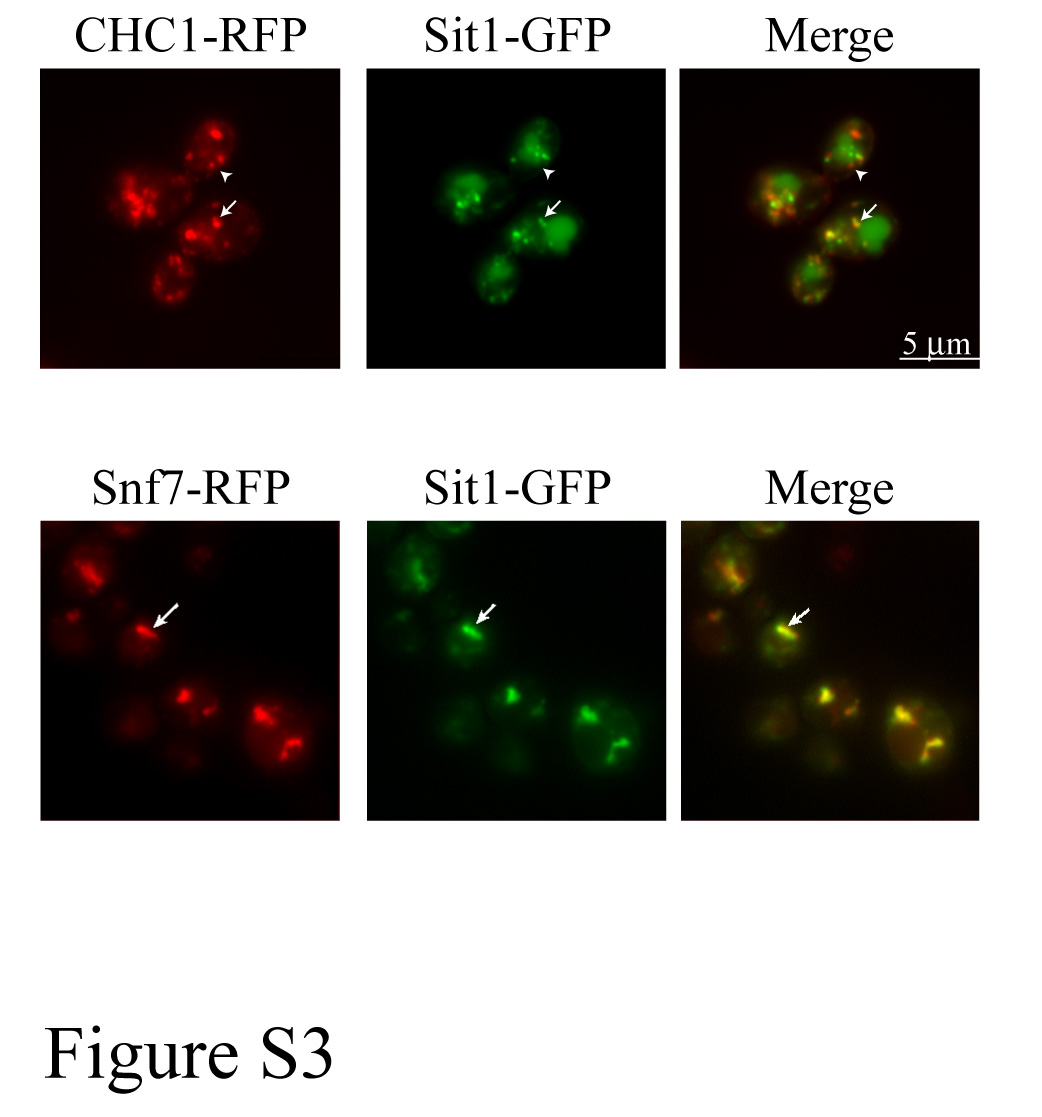

Supplement: Figure S3 — Colocalization of Sit1-GFP with CHC1-red fluorescent protein (RFP) and Snf7-RFP. CHC1-RFP (late Golgi marker) and SNF7-RFP (late endosome marker) strains transformed with pGAL-SIT1-GFP were grown overnight in raffinose-containing medium. Galactose was then added and the culture incubated for 1 h to induce Sit1-GFP expression. The distributions of Sit1-GFP, CHC1-RFP and Snf7-RFP were compared, using the GFP and rhodamine filter sets. Arrows indicate colocalized structures and arrowheads, structures that were not colocalized. Snf7- RFP expression leads to the accumulation of a class E compartment (large late endosome apposed to the vacuole) in which Sit1-GFP is trapped. [file tra0008-1601-SD3.jpg]

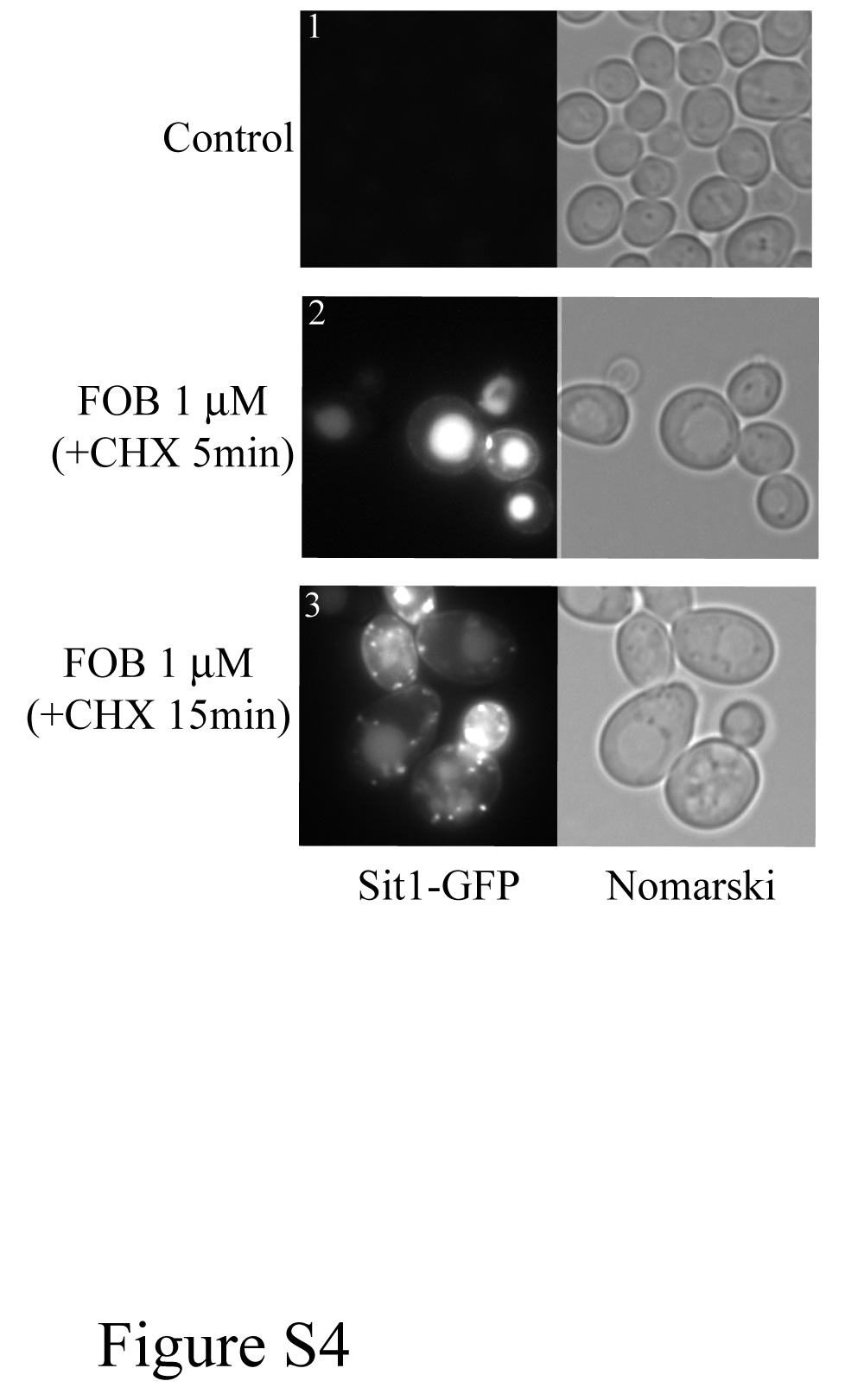

Supplement: Figure S4 — Siderophore-induced relocation of Sit1-GFP independent of protein synthesis. The sit1Δ cells transformed with pGAL-SIT1-GFP were cultured to midexponential growth phase in raffinose-containing medium. Sit1-GFP synthesis was induced by incubation with galactose for 60 min. Protein synthesis was blocked by adding cycloheximide (CHX, 10 μg/mL) to the medium either together with galactose (pannel 1; control to ensure that protein synthesis was immediately blocked with this concentration of CHX) or after the galactose incubation period (panels 2 and 3). In the latter case, cells were incubated with CHX for 5 min (panel 2) or 15 min (panel 3) before adding FOB (1 μm) and incubating the cells for a further 15 min. Sit1-GFP sorting was assessed by fluorescence microscopy, using the GFP filter set. [file tra0008-1601-SD4.jpg]

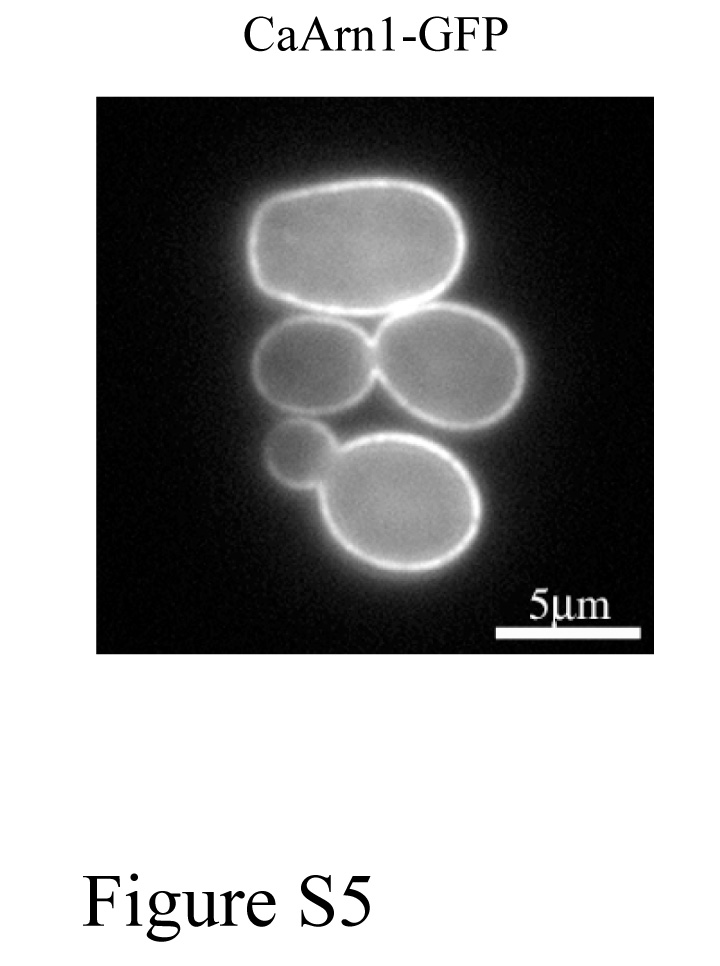

Supplement: Figure S5 — Constitutive targeting of CaSit1/CaArn1 from C. albicans to the plasma membrane. Cells with chromosomal GFP tags were cultured to midexponential growth phase in raffinose minimal medium. CaSit1/CaArn1-GFP synthesis was induced by incubation with galactose for 90 min. GFP fluorescence was assessed by fluorescence microscopy, using the GFP filter set. [file tra0008-1601-SD5.jpg]
